# The role of unintegrated DNA in HIV infection

**DOI:** 10.1186/1742-4690-8-52

**Published:** 2011-07-01

**Authors:** Richard D Sloan, Mark A Wainberg

**Affiliations:** 1 McGill University AIDS Centre, Lady Davis Institute, Jewish General Hospital, Montréal, QC, Canada

## Abstract

Integration of the reverse transcribed viral genome into host chromatin is the hallmark of retroviral replication. Yet, during natural HIV infection, various unintegrated viral DNA forms exist in abundance. Though linear viral cDNA is the precursor to an integrated provirus, increasing evidence suggests that transcription and translation of unintegrated DNAs prior to integration may aid productive infection through the expression of early viral genes. Additionally, unintegrated DNA has the capacity to result in preintegration latency, or to be rescued and yield productive infection and so unintegrated DNA, in some circumstances, may be considered to be a viral reservoir. Recently, there has been interest in further defining the role and function of unintegrated viral DNAs, in part because the use of anti-HIV integrase inhibitors leads to an abundance of unintegrated DNA, but also because of the potential use of non-integrating lentiviral vectors in gene therapy and vaccines. There is now increased understanding that unintegrated viral DNA can either arise from, or be degraded through, interactions with host DNA repair enzymes that may represent a form of host antiviral defence. This review focuses on the role of unintegrated DNA in HIV infection and additionally considers the potential implications for antiviral therapy.

## Review

### Multiple forms of unintegrated DNA

The retrovirus family is characterized by reverse transcription of the viral RNA genome to cDNA and its integration into the host cell genome. Integration of the reverse transcribed cDNA is mediated by the viral encoded and imported integrase enzyme. Integrase excises a dinucleotide from the 3' terminus of the cDNA in a step known as 3' processing. 3' processed viral DNA is then covalently linked to host DNA in a process known as strand transfer [1]. Single stranded DNA breaks, in the host genome at the site of integration, are then repaired by host factors [2]. The viral genome is preferentially integrated into transcriptionally active open chromatin [3-5], following the transcription of viral genes which occurs via host transcription factors, leading to synthesis of the viral transactivating protein, Tat, and subsequent Tat mediated transactivation of the viral LTR promoter. This process ensures that viral genes integrated in the host genome are transcribed, ultimately leading to synthesis of viral proteins and completion of the viral replication cycle [2].

**Figure 1:**
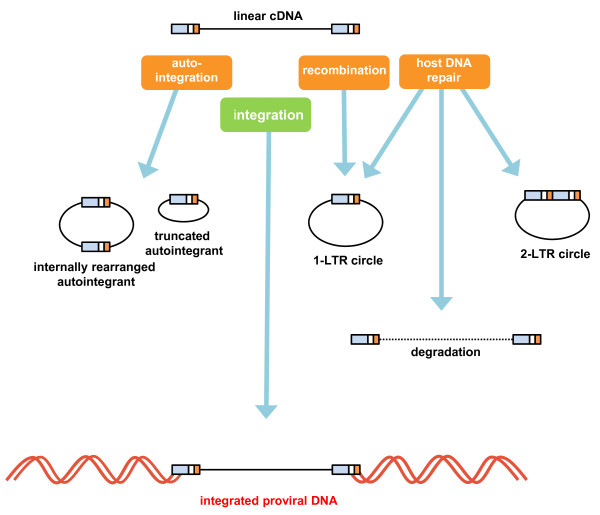
**The various forms of unintegrated HIV cDNA**. Linear cDNA, the product of reverse transcription, is susceptible to a number of fates other than integration into host chromatin as proviral DNA. Autointegration may lead to the formation of truncated or internally rearranged circular forms. Although recombination may yield 1-LTR circles, host factors may also contribute their presence. Host factors, such as those involved in the non-homologous end joining pathway, participate in the formation of 2-LTR circles. Various DNA repair factors and restriction factors may also result in direct degradation of linear cDNA. Collectively, these processes help to explain patterns of unintegrated viral DNA present in infected cells.

However, during natural HIV-1 infection the vast majority of viral cDNA exists in an unintegrated state [6-10]. Multiple forms of unintegrated viral DNA exist, including linear cDNA, the most abundant form that is the direct product of reverse transcribed viral RNA and is the substrate for the integration reaction [6]. All other unintegrated DNA products derive from linear cDNA and are circular in form (Figure 1).

Unintegrated circles can be produced through autointegration (sometimes called suicidal integration), in which the 3'-ends of the reverse transcript are processed by integrase and then attack sites within the viral DNA, producing either internally rearranged or less than full length DNA circles (Figure 1) [2,11]. Autointegration is seen in murine Moloney leukemia virus (MoMLV), Rous Sarcoma Virus (RSV) and HIV-1 infections, and is thus a likely common feature of retroviral replication [12-14]. This process occurs with relatively high frequency, and so approximately 20% of the circular DNA products were found to be autointegrants in MoMLV infections [12].

1-LTR circles are found exclusively in the nucleus and can be formed through homologous recombination of linear DNAs at the LTRs, resulting in a circular DNA bearing one copy of the viral LTR (Figure 1). Early experiments determined that cellular factors were required to mediate 1-LTR circle formation [15]. Later analysis showed that the RAD50/MRE11/NBS1 nuclease components were implicated in 1-LTR circle formation [16]. However, 1-LTR circles can also be formed via ligation of interrupted reverse transcription intermediates (Figure 1) [17]. Interestingly, Foamy virus particles, which can complete endogenous reverse transcription in the virion prior to infection, have been shown to contain 1-LTR circles [18]. In HIV, however, endogenous reverse transcription does not occur naturally, and even *in vitro *assays do not yield near full-length products, so it is unlikely that HIV 1-LTR circles could form outside the cell [19]. In this regard, it must be noted that 1-LTR circles are also absent in the cytosolic fraction of HIV-infected cells [15]. Formal quantification of 1-LTR circles via quantitative polymerase chain reaction (qPCR) is technically challenging, due to a lack of unique sequence features, although end point blot and PCR analysis methods do exist for detection of 1-LTR circles [20,21].

The elucidation of the rolling circle hypothesis of phage DNA replication was formulated in 1968 [22,23], and led to the appealing hypothesis that 2-LTR circles, that contain the full length HIV DNA and both sets of LTRs, might be the direct precursor of integrated DNA (Figure 1). Although some experiments suggested that 2-LTR circular DNA could bind cellular target DNA [24], this hypothesis has since been disproven, and it is now established that linear cDNA is the only precursor to proviral DNA [25-27]. Accordingly, unintegrated circular products cannot sustain replication in themselves and have been considered to be the "dead end products of abortive infections" [2,28,29].

It is now known that 2-LTR circles are the products of non-homologous end joining (NHEJ) DNA repair events that are mediated in the nucleus as a protective host response to the presence of double stranded DNA [10,11] (Figure 1). It has been seen that viral cDNA replication intermediates are associated with host Ku components of the NHEJ pathway [30-32]. Additionally, inactivation of the NHEJ components Ku, ligase 4 or XRCC4 leads to reductions in 2-LTR levels upon infection, whilst inhibition of the DNA-dependent protein kinase catalytic subunit (DNA-PKcs), which is also a component of the NHEJ machinery, had a more modest but measurable effect on 2-LTR circle formation [16,32]. When specific NHEJ processes were abolished in some studies, apoptosis was seen in infected cells [30,33]. Under these circumstances, reverse transcription but not integration was required to yield apoptosis, implicating unintegrated viral cDNA as a key signal that promotes apoptosis when NHEJ processes are depleted [30].

It was previously considered that the cytopathic effect of HIV might actually be due to excessive accumulation of unintegrated cDNAs upon superinfection, as their presence would trigger apoptosis even in infected cells with intact NHEJ machinery [34-36]. But cytopathic effect has since been proven to be separable from accumulation of unintegrated DNA [37,38].

Given that 2-LTR circles are exclusively found in the nucleus, they have become a useful marker of viral nuclear import in studies of viral trafficking [39]. This is due to the unique nature of the LTR-LTR junction that can be readily assayed by PCR [40]. Thus, levels of 2-LTR circles are often recognized as overall markers of total unintegrated DNA in the cell, despite the fact that 2-LTR circles are present at relatively lower levels than other unintegrated DNA species [15,40]. However, detection sensitivity of 2-LTR circles (and other non-integrated forms) can be improved by separating high molecular weight mass genomic DNA from samples [41-43].

### Host cell factors that inhibit viral integration

Other than circularization by NHEJ machinery resulting in 2-LTR circles, there are many further mechanisms that recognize and neutralize infecting retroviral DNA. These involve a variety of factors, many of which are involved in cellular DNA repair processes. For example, XPB and XPD are cellular DNA helicases that are components of the TFIIB basal transcription complex that plays a role in DNA nucleotide excision repair [44]. Recently, XPB and XPD also were implicated in controlling retroviral infection [45,46]. In comparison to cells which have reduced XPD and XPB function, it was shown that retroviral cDNA is degraded in wild type cells in the absence of an accumulation of 2-LTR circles. This implies an XPB- and XPD-mediated mechanism of linear viral cDNA degradation. Further analysis has shown that XPB-mediated degradation of retroviral cDNA is dependent on nuclear entry. However, these restrictive effects do not involve XPB and XPD mediated up-regulation of host gene expression or induction of APOBEC3G or other proteasome-mediated pathways [46].

There are similar findings involving other DNA repair mechanisms; Rad18 is a component of the post-replication DNA repair pathway which was identified as contributing to HIV integrase stability [47]. More recent analysis demonstrated that cells lacking Rad18 were hyper susceptible to infection by MLV and HIV [48]. This effect was even seen with non-integrating virus, leading to the conclusion that Rad18 perhaps exerts its influence on viral cDNA prior to integration. Another example of the involvement of DNA repair pathways in preventing retroviral infection is found in the homologous recombination (HR) DNA repair protein Rad52 [49]. In cells with reduced Rad52 expression, increased levels of HIV-1 transduction were observed upon infection, yet reductions in levels of other HR components (XRCC2, XRCC3 and BRCA2) had no such effect. Interestingly, 2-LTR circle levels were found to be reduced in infected cells that over-expressed Rad52, yet there was no apparent effect on apoptosis. These observations imply a direct degradation of linear viral cDNA by Rad52.

The well characterized restriction factors APOBEC3G and APOBEC3F may also influence the forms of unintegrated DNA seen upon HIV infection. APOBEC3G and APOBEC3F are nucleic acid editing enzymes which restrict viral replication by introducing cytidine to uracil changes in first strand synthesis of viral DNA, resulting in mutated virus [50]. APOBEC3G and APOBEC3F are also thought to function more directly by inhibiting viral reverse transcription, and there now is also evidence that APOBEC3G and APOBEC3F also directly inhibit integration by modifying the linear cDNA substrate, thus rendering it unsuitable for provirus formation [51,52]. APOBEC3G generates a 6 base extension at the U5 end of the viral 3' LTR which causes the linear cDNA to be a less suitable substrate for integrase, whereas APOBEC3F, which has a more potent affect upon integration, functions by inhibiting the 3' processing of the viral cDNA prior to integration. Curiously, APOBEC3G-mediated inhibition of integration leads to a two-fold reduction in 2-LTR circles upon infection with a Δ-*vif *virus when compared controls lacking APOBEC3G [53]. It is possible that the inhibition process may render the linear cDNA template a less suitable substrate for the cellular NHEJ machinery leading to less 2-LTR circle formation, and/or there may be a direct degradation of the modified cDNA.

Another DNA repair factor, uracil DNA glycosylase 2 (UNG2), which is part of the uracil base excision repair pathway, is thought to directly inhibit retroviral DNA at a preintegration step [54], a process which may be counteracted by HIV-1 Vpr [55]. Yet, the precise role of UNG2 in the HIV lifecycle remains controversial; some evidence suggests that UNG2 may be required to mitigate APOBEC3G restriction in order to allow successful reverse transcription [56], but there is also evidence that indicates a lack of involvement of UNG2 in APOBEC3G-mediated effects on infectivity [57]. Recent data also suggests that HIV DNA tolerates a high rate of uracilation, rendering it a poor target for strand transfer when compared to uracil-poor chromosomal DNA, a process which seems to protect viral DNA from autointegration [58]. These contradictory findings make it difficult to reconcile the true role of UNG2 in HIV replication.

Accordingly, multiple host factors involved in DNA repair serve to subvert retroviral infection, resulting in the formation of retroviral cDNA circles. Additionally, other DNA repair mechanisms directly degrade or modify viral linear cDNA and may act in conjunction with constituents of the intrinsic/innate immunity responses, in order to prevent viral integration. The importance of these restrictive measures to the host cell is demonstrated by the finding that NHEJ genes in both yeast cells and primates were under strong selective pressure, indicating a competition between host and pathogen [59,60]. Collectively, these processes help to explain the observation that the majority of reverse transcribed DNA does not obtain the status of integrated viral DNA [61,62].

### Host cell factors that aid viral integration

HIV uses cellular host factors to increase the likelihood of successful integration. One of the best characterized is LEDGF/p75 which is required to tether viral DNA to host chromatin in association with integrase, and also aids virus to preferentially integrate in open chromatin [63-65]. Blocking the integrase-LEDGF/p75 interaction with small molecule inhibitors leads to elevated levels of 2-LTR circles [66]. The host factor HMG I(Y) has been shown to be a component of the pre-integration complex (PIC) for both HIV-1 and MoLV. Although HMG I(Y) can stimulate integration *in vitro*, cells depleted of HMG I(Y) were not defective in regard to HIV infection [67-70]. Another factor which aids integration is the host protein INI 1, also known as SNF5. INI 1, is a core component of the ATP-dependent chromatin remodelling complex SWI/SNF and is also a component of the PIC which can stimulate HIV-1 integrase activity in nucleosome regions of chromatin [71,72]. Thus, multiple host factors are components of the PIC and act in concert to promote the success of the integration reaction; it is possible that more such factors remain to be identified.

Once the integration reaction has been completed, cellular DNA repair enzymes are thought to be used to repair the strand break after the viral genome has been tethered to that of the host. Although the data available provide a far from complete picture, members of the PIKK family, i.e. ATM, DNA-PKcs and ATR have all been implicated in this process [33,73,74]. However, some studies found no influence on HIV-1 transduction when ATM, ATR, DNA-PKcs, and PARP-1 were knocked down [75]. Surprisingly, DNA-PKcs when knocked down led to slightly lower levels of 2-LTR circles, meaning that DNA-PKcs has been described to have both a positive and negative effect on the integration process [16,33]. Although Ku70 depletion can lead to reductions in 2-LTR circle formation, it has also recently been suggested that Ku70 also protects viral integrase from ubiquitination and subsequent degradation, or that Ku70 may be involved in DNA repair after integration of viral DNA into host chromatin, suggesting a positive role for Ku70 in HIV replication [32]. In order to identify novel host factors required for successful integration, an siRNA screen was recently performed that targeted components of cellular DNA repair mechanisms [76]. This process identified proteins involved in base excision repair (BER) as factors required for efficient lentiviral, but not gamma retroviral, integration. Further analysis of this screen characterized the role of the damage recognition glycosylases OGG1 and MYH and the late repair factor POLβ as ones that can augment lentiviral integration, although the mechanistic basis for this is as yet unknown, the authors propose that BER proteins might help to complete repair of the integration intermediate [77].

Retroviruses may also use host factors to increase the efficiency of integration, by reducing the likelihood of autointegration. For MoMLV, the host-derived barrier to autointegration factor (BAF) was found to be a component of the PIC which protects viral cDNA from autointegration [78]. *In vitro *analyses of HIV-1 PICs also found that BAF also functioned in this manner [79]. However, despite clear *in vitro *activity, for HIV the knockdown of BAF in cells did not seem to prevent viral replication [80]. HIV-1 and HIV-2 also use components of the endoplasmic reticulum-associated SET complex, which consists of three DNAses (APE1, TREX1, and NM23-H1), to prevent autointegration. Knockdown of these components measurably increased levels of viral autointegrants following infection [13]. Little is understood about the process, but a direct interaction between the SET complex and the PIC was observed. However, this effect did not extend to either murine leukemia virus (MLV) or avian sarcoma virus (ASV). Given the propensity for retrovirus to autointegrate, it will be interesting to uncover what methods viruses have evolved to counteract this process.

Thus, viral cDNA undergoes a series of complex positive and negative interactions with host factors during integration into host chromatin. These interactions ultimately dictate the levels and proportions of unintegrated DNA species that are observed upon retroviral infection by either influencing the likelihood that certain unintegrated DNA species are formed, by promoting degradation of unintegrated DNA species, or by promoting the likelihood that linear cDNA becomes provirus (Figure 1).

### Transcription of viral genes from unintegrated HIV DNA

The primary function of unintegrated DNA in the HIV replication cycle is to provide the link between viral RNA and integrated proviral DNA, in the form of linear cDNA [2]. Yet, when viral integration may not yet have occurred, transcription of viral genes can still be observed [81,82]. Some experiments have used integrase-defective viruses, in which various point mutations were inserted into the amino acids of the catalytic triad D(64)D(116)E(152), to yield a non-functional integrase domain of the pol polyprotein which becomes packaged into an otherwise functional virion [83]. Common mutations for this approach are D64E, D116N and E152A, but inhibitory concentrations of integrase strand transfer inhibitors, such as raltegravir, can also be used to block integration [84].

**Figure 2:**
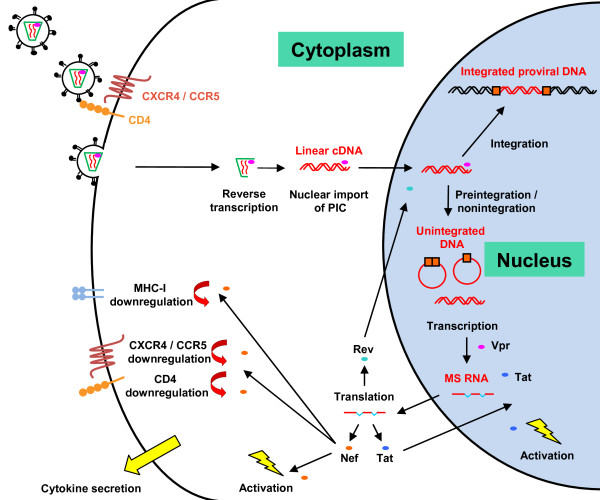
**Transcription from preintegrated or unintegrated DNA**. Prior to integration, or if integration is blocked, transcription from unintegrated cDNA may still occur, the template for which is unknown. Virally imported Vpr is important in the initial stages of viral gene transcription. Translation of multiply-spliced RNA (msRNA) transcripts leads to expression of Tat, Nef and Rev. Levels of Rev are insufficient to lead to the export of singly spliced and unspliced transcripts. Rev is thought to later interfere with the integration process and to thereby inhibit superinfection. Tat and Nef collectively lead to increased cellular activation in resting T-cells. Newly synthesized Tat will also promote viral gene transcription. Nef downregulates cell surface CD4, CXCR4, CCR5 and MHC-I (HLA Class I), thereby limiting superinfection, signal transduction and likely resulting evasion from cytotoxic T-lymphocytes. Preintegration transcription of viral genes has also been linked to altered cytokine secretion in both resting T-cells and macrophages.

Using these approaches, it has been shown that virally imported Vpr can promote the transcription of viral genes from unintegrated DNA, a process that is independent of Tat transactivation [85]. This process of Vpr-mediated transcription may ultimately lead to Tat expression and subsequent positive feedback of the transcription process from unintegrated DNA via Tat. Thus, one role of virally imported Vpr may be to initiate transcription and early Tat synthesis (Figure 2).

When transcription from unintegrated DNA does occur, all classes of multiply-spliced, singly spliced and unspliced viral mRNA transcripts can be observed (Figure 2) [86-88]. However, the relative proportions of each splice class vary compared to those observed during productive infection, *i.e. *whilst multiply spliced transcripts are abundant in the absence of integration, levels of singly-spliced and unspliced transcripts are reduced in this circumstance [86,87]. Both integrating and non-integrating virus produced similar levels of multiply spliced viral mRNA transcripts in infections of the Rev-CEM T-cell line when assayed by qRT-PCR [81]. Another study described a transcript unique to the LTR-LTR junction of 2-LTR circles, though it is unknown if this transcript fulfils any function [89].

Despite extensive transcription from unintegrated DNA, a key limitation in the translation of viral genes leading to the expression of late viral gene products is the low levels of Rev that are transcribed from unintegrated DNA. A paucity of Rev limits the nuclear export of Rev-response-element (RRE) bearing-singly-spliced and unspliced transcripts, which code for structural proteins or are incorporated into nascent virions. Providing Rev *in trans *can rescue late gene synthesis [88].

In the case of the Rev-CEM indicator cell line [90], transcription of GFP is under the control of the HIV-1 LTR, and the gene is surrounded by splice donor and acceptor sites downstream of a RRE [91]. This cell line was made by transducing the parental CEM-SS T-cell line with the pNL-GFP-RRE-SA construct. In the presence of Tat, the viral LTR is transactivated and mRNA produced, but, if Rev is absent, the GFP coding sequence is spliced out and not translated. Thus, GFP is expressed in infected cells due to the presence of both Tat and Rev; this is also the case for integrase defective infections, as Tat and Rev can also be expressed from an unintegrated template [92]. As the system is co-dependent on Rev, there is very little transactivation of the viral LTR by cellular factors as occurs with reporters that are dependent only on Tat [90]. The cell line is therefore useful for detecting transcriptionally active viral infections by GFP, even from non-integrated templates, as was seen in a study that characterized the degree of transcription from preintegrated HIV [92]. Previous calculations, based on Tat transactivation of the viral LTR alone in HeLa-CD4-LTR-β-Gal indicator cells, estimated that total transcription from unintegrated templates following infection with integrase defective virus was about 10% of that for productive infections [93]. The Rev-CEM-based study, using a parallel approach, showed that expression from integrase-defective virus was around 70% of that of productive infections [92]. The higher level of LTR transactivation from cellular factors in the earlier study could have resulted in a high background readout that masked detection of some transcripts, a problem avoided with the more specific Rev/Tat co-dependent approach.

The second goal of the study was to address the nature of the transcriptional template in non-integrated infections. It was possible to sort the transcriptionally active cell population bearing unintegrated DNA based on infection-induced GFP expression in Rev-CEM. 2-LTR circle levels were measured by qPCR in the GFP positive cells [92]. Overall, there were many fewer detectable 2-LTR circles than the total number of actively transcribing GFP positive cells. The authors concluded that 2-LTR circles alone could not entirely account for the level of transcription that was seen.

A different study aimed to define the transcriptional capacity of each unintegrated HIV DNA template by constructing artificial linear cDNA, 1-LTR and 2-LTR circle mimics and transfecting each of them into HeLa cells [94]. It was found that all three species of unintegrated DNA could serve as transcriptional templates, and that 1-LTR circles in particular could lead to high levels of viral protein expression. However, all unintegrated HIV DNA forms yielded levels of protein synthesis that were an order of magnitude less than for integrating virus. This finding, combined with the observation that there are relatively high numbers of 1-LTR circles in comparison to the other templates, implies that 1-LTR circles could be a major contributor towards transcription from unintegrated templates [15]. However, this study also noted that late gene products, such as p24, were synthesised from all unintegrated templates. This finding is at odds with studies that assayed transcription from unintegrated DNA via viral infections that yielded no p24 synthesis [88]. This demonstrates that the means of delivery of viral DNA to the nucleus might influence the level of transcription observed; alternatively the cell type may also be a factor [93]. Nonetheless, all three forms of unintegrated DNA have the innate potential to serve as a transcriptional template, raising the question as to why this does not occur to a higher level in infections.

In other studies, expression of late viral genes from SupT1 cells and monocyte-derived macrophages infected with integrase defective virus was augmented through treatment of the cells with short-chain fatty acid histone deacetylase inhibitors [95]. These findings suggest that unintegrated DNA must be contained, in part, in condensed chromatin structures. This was surprising as studies of transfected plasmid DNA had indicated that such constructs would typically be maintained as part of open chromatin, but may be silenced by epigenetic mechanisms over longer time periods in stable transfections [96-98]. This suggests that the presence of viral DNA that has been part of the PIC leads to a specific pattern epigenetic modifications and associations with host factors that are not necessarily captured in transfection studies. These results also imply that there is active control of transcription from unintegrated DNA and it will be interesting to uncover if this influence is due to the virus or the host cell.

The issue of how transcription of viral cDNA arises from unintegrated infections is important, since expression of early viral genes might have benefit for HIV infection. This topic also has implications for gene therapy, since delivery of non-integrating retrovirus to a target cell could lead to expression of genes of interest without the risk of insertational mutagenesis as could occur with integrating vectors. Therefore, understanding and optimising gene transcription from non-integrating lentivirus is an important endeavour [99-102].

### Translation of viral genes from unintegrated DNA

It is now understood that circular unintegrated HIV DNA is not a precursor for viral integration, so it was surprising that one study noted that integrase-defective virus could nonetheless yield synthesis of all viral gene products and to productive infection itself [93]. This led to the proposal that cell-type specific differences might exist in the capacity of cells to sustain transcription from unintegrated DNA. However, such synthesis of late genes from unintegrated DNA was later understood to be only observable in T-cell lines such as MT-2 that were chronically infected with HTLV-1, it was later concluded that the presence of transcriptionally active HLTV was able to rescue integration-defective HIV [103]. However, other studies have also demonstrated that infections of various T-cell lines, activated or resting primary CD4^+ ^T-lymphocytes and macrophages, may lead to expression of a limited range of viral proteins in the absence of viral integration. There is evidence for Tat transcripts from unintegrated DNA [87-89]. However, there is no direct evidence for the expression of Tat, in part due to difficulty in resolving it through Western blot at low levels. There is however much indirect evidence for Tat expression from unintegrated DNA due to its capacity to transactivate viral LTRs [82,93]. The same is true for Rev, although Rev transcripts have been readily identified from non-integrated infections [88], there is no evidence directly showing Rev expression in this circumstance. Nonetheless, its expression can be readily inferred from Tat and Rev dependent Rev-CEM GFP reporter cells which express GFP even when infected with integrase defective virus [90,92]. Nef is the only viral protein that can be readily demonstrated to be expressed from non-integrated viral infections, and has been observed in a number of studies [81,87,88,104].

Tat has a role in modulating T-cell activation, and it has been shown that expression of Tat and Nef from unintegrated DNA in resting T-cells increases cellular activation, IL-2 secretion and the likelihood of productive infection (Figure 2) [86]. These data show that expression of viral genes prior to integration can assist the infection process. It is still unclear if the fate of every PIC imported into the nucleus is to perform this function in order to prime cells for successful infection, but it is a very appealing concept.

Patterns of transcription and translation prior to integration in productive infections of T-cells are identical to those seen in the absence of integration [88]. This suggests that studies of gene expression in which integration has been blocked are equivalent to studies of gene expression prior to integration. Experiments that use common mutations in the integrase DDE catalytic triad or that employ integrase inhibitors to prevent integration, may therefore model preintegration events.

The best-studied HIV protein in this context is Nef which is a multifunctional non-enzyme adaptor protein that acts to subvert cellular signalling and trafficking pathways [105]. As Nef is myristolated, it is directed to cellular membranes, where it exerts many of its roles in immune-evasion, cellular activation, and modulation of virion infectivity [106,107]. The first two of those roles indicate that it is advantageous that Nef be expressed early in infection for viral replication. In support of this, Nef-mediated functions are present even in the absence of viral integration [81,86].

In addition to modulating the activation threshold of infected CD4^+ ^resting T-cells, Nef can downregulate cell surface CD4 expression in activated primary CD4^+ ^T-cells infected with integrase-defective virus [108]. Another study confirmed Nef-mediated downregulation of CD4 in the SupT1 cell line, and further demonstrated that this activity was predominantly dependent on the import of Vpr with the virion in order to promote the initiation of transcription [109]. In studies using the Rev-CEM cell line, it was seen that Nef, expressed in the absence of integration, could downregulate each of the chemokine co-receptors CCR5 and CXCR4, and CD4 [104]. Thus, the products of unintegrated DNA can promote extensive downregulation of entry receptors (Figure 2). This process might be to restrict superinfection and its associated toxicity. Indeed, Nef can restrict superinfection via downregulation of CD4, CCR5 and CXCR4 during productive infections [110-112]. An additional benefit might extend to a reduction of signal transduction through these receptors which might otherwise affect transcription, chemotaxis and apoptosis [113-115]. Whilst signal transduction following viral binding to coreceptors is important in infection [114], excessive additional signalling after entry might interfere with infection.

Rev may interact with viral integrase and the host factor LEDGF/p75 to negatively regulate integration [116,117]. This is seen with both integrating and non-integrating virus, thereby effectively regulating superinfection at the level of integration rather than entry [117]. Expression of Rev might not significantly inhibit the first infecting and Rev producing virus, but might inhibit further superinfecting viruses from integrating. The authors of these studies also demonstrated that entry receptor downregulation contributed to restriction of superinfection prior to integration, as additional superinfection resistance was seen with following infection with a Δ-*rev *virus bearing an HIV envelope when compared to a Δ-*rev *VSV-G envelope bearing pseudovirus. Such findings are consistent with studies showing that downregulation of CD4 and chemokine receptors reduces superinfection [104,108,109], and is also consistent with studies that use an inducible cell line (293-Affinofile) to control receptor and coreceptor density in order to demonstrate that their reduction leads to proportional loss of infection [118-120]. Thus, Rev and Nef can act in concert to restrict superinfection prior to, or without, integration (Figure 2).

Nef also has a role in immune evasion by inducing downregulation of the human leukocyte antigen (HLA) class I allotypes that are recognized by cytotoxic T-cells (CTLs), i.e. HLA-A and HLA-C, while selectively not downregulating HLA antigens recognized by NK cells (HLA-B and HLA-E), which could respond to downregulation by inducing apoptosis [121-124]. Studies of infected Rev-CEM cells showed that Nef expressed from unintegrated virus could downregulate HLA-ABC (*i.e. *an epitope composed of HLA-A, HLA-B and HLA-C in combination), HLA-A31, but not HLA-E, essentially mirroring the effects seen in productive infections [81]. The extent of downregulation seen in the absence of integration was similar to that seen in productive infection using wild type virus. Thus, the activity of Nef was not linked to integration in regards HLA class I modulation, a finding confirmed in primary activated CD4^+ ^T-cells. This is also consistent with current understanding that CTL responses are an important contributor in immune control of HIV infection [125-127]. Thus, another benefit of early Nef expression may be immune evasion from CTLs for virus that has not yet integrated.

For cell types with slower replication kinetics the lag between initiation of transcription from preintegrated DNA and transcription of provirus might be long, providing a larger window of benefit for products of unintegrated DNA in regard to immune evasion. In macrophages, integration of the viral genome can take 2-3 days [128], although maximum integration levels in a cell culture population required as many as 30 days [87]. In resting CD4^+ ^T-cells, this process can take 2-3 days [86], whereas for activated CD4^+ ^T-cells or T-cell lines, an average of only 12-24 hours is required [129]. In the case of resting CD4^+ ^T-cells, however, there may be limitations on nuclear export of multiply-spliced viral transcripts [130], although there is evidence of gene expression in this state [82,86]. Thus in all HIV-1 infections the only viral DNA is unintegrated over a significant period of time. It may be that the transcription observed during this period is beneficial. Therefore, the role of Tat, Nef and Rev regarding their many other functions, but prior to integration, is unknown and therefore remains an interesting question [105].

### Persistence of unintegrated DNA in infected cells

Although other viral episomes (e.g. hepatitis B virus (HBV) covalently closed circular DNA (cccDNA) [131,132] and herpesvirus episomes [133,134]) can be stable within host cells, unintegrated HIV DNA lacks an origin of replication; and so it is not copied with each cell division. Additionally, linear unintegrated cDNA is more labile than circular forms inside cells [88,135]; this pattern may be explained by host defence and DNA repair responses directed to the presence of linear cDNA. The ultimate stability of circular cDNA forms, which are generally stable in cells, is then therefore largely driven by the rate of cell division [136-138]. Accordingly, a rapid rate of lymphocyte turnover and cell division explain why 2-LTR circle levels are not well maintained in the total CD4^+ ^T-cell population in patients [138], despite cell culture data demonstrating their relative intracellular stability [136]. Maintenance of circular HIV cDNA in dividing cells can be rescued when an origin of replication is introduced into integrase-defective HIV [99,139]. Further, experiments that sought to arrest the cell cycle of T-cells through use of cell cycle inhibitors such as aphidocolin, which arrests cells in the G1/S phase, also demonstrated that unintegrated DNA circle stability was increased to ≈ 5-7 days in such cells [136,140-142].

Infections of non-, or slowly-dividing cells can occur *in vivo *(e.g. naïve CD4^+ ^T-cells, resting memory CD4^+ ^T cells, and macrophages). In infections of quiescent CD4^+ ^T-cells, reverse transcription can occur, but is often not completed and displays greatly reduced kinetics, or PICs might not be imported into the nucleus efficiently when levels of ATP are lacking; therefore integration can be delayed or may not occur at all [86,143,144]. In these circumstances, unintegrated DNA may persist in the resting cell, and viral gene transcription may be observed [82]. Subsequent activation of the cell prior to degradation of the functional PIC may yield productive infection; hence this state is referred to as preintegration latency [10,144-149]. This form of latency is therefore more labile and functionally quite distinct from post-integration latency that can happen when integration occurs, but the provirus is transcriptionally silent, an outcome that can be rendered through a variety of host-mediated mechanisms [144].

Experiments in macrophages, which are a naturally non-dividing population, have also demonstrated longevity of unintegrated DNA. One study found that macrophages infected with integrase-defective virus still contained cells bearing unintegrated DNA up to 30 days post-infection [87]. Viral mRNA transcripts were detectable throughout as were viral proteins such as Nef. A similar study on infected macrophages performed with an integrase defective virus, bearing a luciferase reporter gene showed that unintegrated DNA products were still detectable in the cell up to 21 days post infection; luciferase was detectable throughout the study period [150]. Finally, infections of animal models with integrase defective lentiviral vectors for gene therapy studies found that such vectors were very stable in non-dividing cells for extended periods, up to one year in some instances [151,152]. Therefore, unintegrated HIV-1 DNA likely has the capacity to persist in slow or non-dividing cells *in vivo*.

### Unintegrated DNA as a diagnostic marker

There has been interest in using 2-LTR circle titres as measured by qPCR as a clinical diagnostic assay, since it was hoped that their levels would be representative of nascent infections [8]. This approach was supported by prior observations that levels of total unintegrated DNA decrease during highly active antiretroviral therapy (HAART) [153,154]. These findings can be explained by degradation of abundant linear unintegrated cDNAs within cells and dilution of circular forms with each cell division [6]. Subsequent studies of HIV-infected patient samples demonstrated that measuring 2-LTR circle levels specifically was not a reliable marker of effective therapy when compared to plasma viral RNA [43,135,141]. Confounding factors for this approach are likely due to the persistence of 2-LTR circles in long lived or non-dividing cellular reservoirs, the lag between administration of antiviral drugs and actual blockage of infection, and the possibility of ongoing replication, or viral release from stable reservoirs despite seemingly effective therapy [87,135,155].

Some of these potentially mitigating effects have been investigated by monitoring 2-LTR circle levels in patients during drug intensification studies in which further drugs are added to an already successful highly-active antiretroviral therapy (HAART) regimen. In one such study, patients with undetectable viral load were given the integrase inhibitor raltegravir [156]. In these circumstances it is argued that the detection of an increase in 2-LTR circle levels is indicative of *de novo *viral infection that continues in the face HAART, but below the detection limit of quantification of common qRT-PCR assays. Evidence was found for a surge of 2-LTR circles in 13/45 (29%) patients upon intensification, yet this did not translate to a change in plasma viral RNA levels when using a sensitive single copy assay. This latter finding was confirmed in a randomized clinical trial wherein again no decrease in plasma viral RNA was seen with raltegravir intensification [157]. Given the contradictory nature of these findings, it is unclear to what extent raltegravir intensification does inhibit ongoing infection and why an apparent inhibition of replication does not go on to alter viral load. One suggestion is that the cells in which there is a 2-LTR surge arise from a site which does not communicate freely with plasma [157]. However, it should be noted that a previous small scale study of treatment intensification using non-nucleoside reverse transcriptase inhibitors (nnRTIs), or protease inhibitors, found that adding these drugs had no effect on viral load using single copy qPCR assays, implying that ongoing infection is likely not the source of residual viremia [158]. This concept is supported by phylogenetic evidence showing that upon treatment interruption rebounding virus arises from a small number of invariant clones, a finding that does support the notion of ongoing replication [159].

Yet having knowledge about levels of 2-LTR circles might still provide clinically useful data. A recent study isolated *env *sequences from 2-LTR episomes in patients who suspended therapy [160]. It was shown by sequence analysis that rebounding virus matched that found in viral episomes prior to plasma viral RNA rebound. Thus, episomal sequences might predict the potential for emergence of resistance mutations or altered coreceptor tropism. Therefore, although the value of knowing 2-LTR circle levels in therapy has been discredited and is also disputed in intensification studies, sequences deriving from such circles may still be of clinical benefit.

Further, knowing 2-LTR circle levels can still provide useful data in clinical and pre-clinical studies in which integrase mechanisms are being studied. For example, elite suppressors of HIV, *i.e. *patients who control their infection successfully without antiviral therapy, were found to have lower rates of viral integration and higher levels of 2-LTR circles than observed in patients who were on or off HAART [161]. The mechanism underlying this effect is unknown, but *ex vivo *analysis has excluded a role for innate restriction factors that affect viral integration. A more recent study of CD4^+ ^T-cells from elite controllers suggests that upregulation of cellular p21 in such cells might be important in how they resist infection, but the effects of p21 were seen at the level of viral gene transcription and not at integration [162]; therefore, the factor that might underlie any integration-related effects remain to be identified.

### Unintegrated viral DNA and antiviral therapy

Since the development of the first integrase strand transfer inhibitors, it has been known that their use leads to elevated levels of unintegrated DNA as measured via qPCR for 2-LTR circles [40,84]. In the absence of integration, there is greater substrate availability for the cellular NHEJ pathway [30]. This phenomenon has been of utility in cell culture studies of integrase inhibitor therapy, as levels of 2-LTR circles, relative to levels for wild-type virus, can be considered to be indicative of integrase dysfunction [163,164].

The observation of elevated 2-LTR circle levels with integrase inhibitors has led to some speculation that these might influence the natural course of infection or the success of therapy. In clinical trials, use of the integrase strand transfer inhibitor raltegravir, compared to the non-nucleoside reverse transcriptase inhibitor efavirenz [165-167], led to more rapid viral RNA decay kinetics [166]. One study suggested that increased apoptosis in HIV-1 infected cells, due to accumulation of unintegrated DNA, might explain these kinetics [168]. However, an alternative explanation, based on mathematical modeling of the rate of viral decay in the various infected cell types is that raltegravir acted at a later stage of viral replication than efavirenz, and was thus able to influence its antiviral effect on a larger population of infected cells [128,169,170]. Confirmation of this model was achieved in cell culture analysis, which demonstrated that the stage of viral replication targeted by each drug class contributes to the effectiveness of viral RNA decay. Furthermore, the success of each drug combination was controlled by the latest acting drug in the combination [129].

Despite their effectiveness, integrase inhibitors are unique in their capacity to lead to populations of cells in being able to block replication at an early stage with reduced cytopathic effect. Although expression of early viral gene products in this circumstance is observed, integrase inhibitor treated cells block infection in such a way that the cell cannot directly contribute to viral load. However, in patients receiving raltegravir treatment, a surge in 2-LTR circle proliferation is seen following therapy [171]. This effect is only temporary in PBMCs, as they lose unintegrated DNA with cell division [136,138]. But it remains to be seen for how long unintegrated DNA might persist in slow or non-dividing cell types in patients receiving integrase inhibitor therapy, given data for infections with integrase defective lentiviral vectors in animal models, it might be anticipated that such a reservoir would be relatively long lived [151,152].

The persistence of unintegrated cDNA is important since infection in cells infected by integrase-defective viruses can be rescued by a superinfection with wild type virus [172,173]. The second, integrating infection can yield Tat to promote transcription from the unintegrated template, resulting in synthesis of full length genomic RNA from the unintegrated DNA. Such RNA will be packaged into virions, providing opportunity for recombination also [172]. These findings mirror early observations on viral replication with integrase defective viruses, suspected to result from HTLV-1 complementation [93]. Similar observations have been made with integrase-competent drug resistant virus, in which drug sensitive virus has been rescued by drug resistant virus in cell culture [174], so there is little reason to suspect that this could not occur with integrase inhibitor resistance and unintegrated DNA. In this sense, both unintegrated and integrated viral DNA can be considered to be viral reservoirs [175].

### Conclusions and Perspectives

Without integration, virus cannot initiate late gene synthesis and productive infection [28,29]. Even with successful entry and reverse transcription, there is a rate of attrition associated with attempts to integrate virus into host chromatin that is mediated by host factors [61,62]. Of the host mechanisms identified in this process, the NHEJ pathways have perhaps been better characterized [16,30], but very little is understood about the apparent direct degradation of linear DNAs by factors such as the cellular DNA helicases XPB and XPD [45,46]. It is still not known how HIV overcomes the obstacles of DNA repair and host-restriction factors directed against pathogen DNA; as despite their function, viral integration still readily occurs in target cells. These interactions are likely complex. For example, the cellular nuclease Trex1 is important in controlling endogenous retroelements by metabolizing reverse transcribed DNA [176,177]. Conversely, HIV-1 has been found to use Trex1 to digest the non-productive DNA by-products of reverse transcription in order to evade host nucleic acid sensing proteins and subsequent triggering of innate immunity pathways [178]. Such DNA detection may lead to apoptosis of the infected cell, a process which may underlie CD4^+ ^T-cell depletion of lymphoid tissue [179]. The relative importance of these host-pathogen-DNA interactions is demonstrated by the unexpected finding that primate NHEJ genes are under strong positive selection [60]. Detailed understanding of such defence pathways will have important consequences for understanding how the host tolerates DNA-utilizing viruses.

Persistence of unintegrated viral DNA in the nucleus can yield extensive transcriptional activity, either before, or in the absence of integration [81,180]. Expression of certain gene products early in the viral life cycle could provide an advantage to the virus e.g. effects on T-cell activity via Tat and Nef [86], as well as downregulation of CD4, CXCR4 & CCR5 via Nef [101,102,106], modulation of HLA Class I expression via Nef [81], and restriction of superinfection at the level of entry and integration via Nef and Rev respectively [117]. It is unclear whether all viruses perform preintegration transcription, as this cannot be elucidated from studies of cell populations. Kinetic and single cell analyses might help to better define this process.

Despite such functionality, 1-LTR and 2-LTR circles *are *dead end products of failed infections and their demise is mediated either by host factors or by the virus itself [16,30]. Conceivably, unintegrated DNA species are simply transcribed because they are present in the nucleus. Yet, studies of transfected versions of these products show different patterns of gene expression, arguing against this viewpoint [94]. Gene expression from unintegrated DNA seems to be controlled in natural infection. The expression of early gene products suggests a benefit for infection. However, it may be argued that such genes that are the first to be expressed anyway. A possible epigenetic modification of unintegrated DNA is intriguing given parallels with control of gene expression in HBV and herpesvirus episomes [95,181,182]. The nature of the transcriptional template for preintegration transcription is unknown; all DNA species remain candidates, although 2-LTR titres are too infrequent to be the predominant template [92]. This information would have relevance for gene therapy approaches using non-integrating vectors [101].

How frequently might transcription from unintegrated templates occur in lentiviruses? In SIVs, which are closely related to HIV, there might be benefit from a similar pattern of early gene expression [183]. Further afield, it is interesting to consider if viruses which encode different, or more limited, early genes might benefit from preintegration transcription, such as feline immunodeficiency virus (FIV) that does not encode *nef*, but does contain *rev *[184].

Preintegration latency may contribute to viral RNA decay dynamics with therapy, but is likely to play only a minor role [128,148,169,170]. Though it is unknown how long unintegrated HIV DNA can persist in other non-dividing cell types *in vivo*, but the results of extended periods of gene expression in macrophages in cell culture suggest a capacity to persist, over long periods [87,150]. The ability of such unintegrated DNA to be rescued, and perhaps recombine with a second incoming virus, might be a contributing factor to the generation of viral diversity [172]. Drug resistant viruses can also rescue non-resistant viruses and it is likely that unintegrated DNA could equally contribute to diversity in this context [174].

## Conclusions

In summary, much of the true nature and function of unintegrated DNA species still remains enigmatic, but unintegrated DNA may well fulfil a multitude of roles in the promotion of HIV infection.

## Competing interests

The authors declare that they have no competing interests.

## Authors' contributions

RDS wrote the manuscript. MAW modified parts of the manuscript in his role as head of the laboratory. Both authors read and approved the final manuscript.
